# A Review of the Technology, Training, and Assessment Methods for the First Real-Time AI-Enhanced Medical Device for Endoscopy

**DOI:** 10.3390/bioengineering10040404

**Published:** 2023-03-24

**Authors:** Andrea Cherubini, Nhan Ngo Dinh

**Affiliations:** 1 Cosmo Intelligent Medical Devices, D02KV60 Dublin, Ireland; 2 Milan Center for Neuroscience, University of Milano–Bicocca, 20126 Milano, Italy

**Keywords:** colonoscopy, AI, CADe, CADx, polyp detection, lesion characterization

## Abstract

Artificial intelligence (AI) has the potential to assist in endoscopy and improve decision making, particularly in situations where humans may make inconsistent judgments. The performance assessment of the medical devices operating in this context is a complex combination of bench tests, randomized controlled trials, and studies on the interaction between physicians and AI. We review the scientific evidence published about GI Genius, the first AI-powered medical device for colonoscopy to enter the market, and the device that is most widely tested by the scientific community. We provide an overview of its technical architecture, AI training and testing strategies, and regulatory path. In addition, we discuss the strengths and limitations of the current platform and its potential impact on clinical practice. The details of the algorithm architecture and the data that were used to train the AI device have been disclosed to the scientific community in the pursuit of a transparent AI. Overall, the first AI-enabled medical device for real-time video analysis represents a significant advancement in the use of AI for endoscopies and has the potential to improve the accuracy and efficiency of colonoscopy procedures.

## 1. Introduction

Colonoscopy procedures provide an excellent opportunity to develop advanced medical devices that utilize artificial intelligence (AI) [1,2,3,4]. This is primarily because physicians must analyze a large amount of information from a real-time video stream, often working under time constraints and facing repetitive tasks during extended periods. In cases where human decisions may be inconsistent [5], AI has been demonstrated to be a beneficial tool for assisting physicians in making more accurate and reliable decisions [6].

In this context, it is relevant to highlight how the intelligent devices that are used in endoscopy or other medical disciplines and have reached the market have the common aim of assisting the clinician in specific tasks and associated medical decisions [7]. Therefore, all AI-enhanced devices today belong to the “Narrow” AI category, in which the medical device’s intended use is to aid a decision with a specific medical task and a well-defined patient population. This is opposed to the category of “General” AI, which comprises fully autonomous algorithms that are capable of automating multi-dimensional decision making, such as diagnosing a condition, independent of human interpretation or intervention [8,9]. Unfortunately, the promise of fully autonomous algorithms for medical decisions inflated the expectations of the scientific community at the beginning of the AI revolution, leaving scars and doubts about the ability to deliver this promise of a real benefit for the patients that are still present [10].

Nevertheless, the area of the AI-supported interpretation of medical images and videos is now blooming [11], and the algorithms in this arena are conventionally identified [12] as follows:

- CADe (computer-aided detection): algorithms that are able to localize/highlight the regions of an image that may reveal specific abnormalities;
- CADx (computer-aided diagnosis): algorithms that are aimed at characterizing/assessing the disease type, severity, stage, and progression.

**Figure 1:**
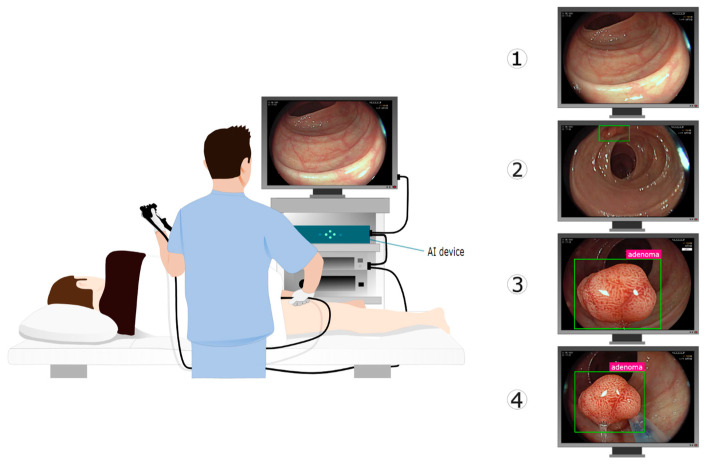
GI Genius is an intelligent endoscopy module designed to be an adjunct to video colonoscopy, to support the operating physician with AI during the colonoscopy. GI Genius augments the video stream by adding graphical overlay markers surrounding areas of interest. As a result, they can be further inspected and assessed independently according to established clinical practice. On the right side, the sequence of actions during a colonoscopy with the assistance of an AI device is depicted: (**1**) endoscope navigation through the colon; (**2**) polyp detection (CADe); (**3**) polyp characterization (CADx); and (**4**) polyp resection. Illustration from Biffi et al. [19].

During a colonoscopy, the endoscopist has various responsibilities and medical choices (Figure 1). While navigating, the endoscopist ensures that the mucosa is suitably exposed, in order to facilitate the detection of polyps. The detection of polyps can be aided by an AI device (CADe) that overlays a marker onto the image region where the AI has detected a potential polyp. Subsequently, the endoscopist examines the mucosa to describe the lesion and chooses the appropriate clinical action. An AI device (CADx) can help to optically characterize this lesion and provide information about the nature of the polyp under examination. Once the polyp has been endoscopically resected, the navigation continues. These tasks are repeated on a loop until the completion of the procedure.

In this work, we provide an overview of the technology and scientific evidence behind GI Genius, the first AI-empowered medical device for colonoscopy to enter the endoscopy market [13,14,15,16,17,18,19,20,21,22,23,24,25,26,27,28,29]. In the following sections, we briefly describe the technical architecture of the hardware and software configuration, the AI algorithm training and testing strategies, and an update of the regulatory path. Finally, we discuss the strengths and limitations of the current GI Genius intelligent device.

## 2. Device Overview

GI Genius is an intelligent endoscopy module that is designed to support physicians during colorectal cancer (CRC) screening and surveillance procedures. It enhances the video stream of a colonoscopy in real time by overlaying graphical markers around the areas of interest, as identified by its CADe algorithm. The device also utilizes a CADx algorithm to provide information about the nature of the abnormalities. GI Genius is developed and manufactured by Cosmo Intelligent Medical Devices, Ireland, which is a part of the Cosmo Pharmaceuticals Group. The medical device, GI Genius, is commercialized and distributed worldwide by Medtronic, Ireland. It is designed to be an adjunct to traditional colonoscopy procedures and does not replace the physician’s role. Instead, it aims to aid the physician in their decision making process and improve the accuracy and efficiency of the procedure.

In addition to the box surrounding the area of interest, if the endoscopist frames the same spot steadily, GI Genius provides a histology prediction in two categories: either adenoma or non-adenoma. A no-prediction message will appear if GI Genius cannot reach a prediction.

GI Genius has been trained to process colonoscopy images that contain regions that are consistent with colorectal lesions such as polyps, including those with a flat (non-polypoid) morphology.

To make GI Genius work, the module is placed on a shelf of a standard endoscopy tower and connected to both the video processor and the main display. As a result, the endoscopy video stream flows through the module without modification or delay, and without changing how the endoscopy procedure is carried out.

### 2.1. Regulatory Status

**Figure 2:**
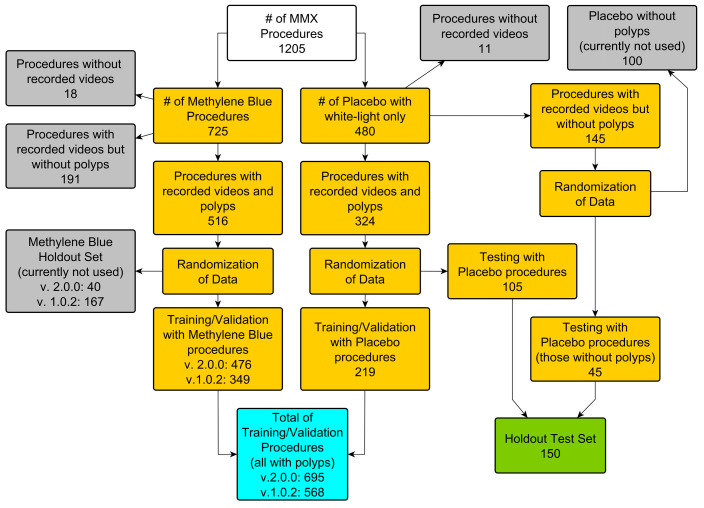
Dataset subdivision for GI Genius CADe v1 and v2. Both versions of CADe have been trained using the data from the international multicenter MB-MMX study (ClinicalTrials.gov NCT01694966 [39]). Both procedures from the methylene blue arm and the placebo arm were recorded using a lossless video compression format. In addition, the holdout test set patients were used for the first standalone CADe performance assessment [14].

The GI Genius module reached different markets at different times due to the various regulatory paths for AI-enhanced medical devices [30,31]. The first GI Genius feature to reach the market was version 1 (v1) of the CADe module for polyp detection. CADe v1 was introduced in Europe with the CE mark in 2019. It was used in a randomized controlled trial that studied the effectiveness of GI Genius for improving the adenoma detection rate [15]. In 2020, version 2 (v2) of the CADe, with improved detection performances, was distributed in Europe. These enhanced performances were mainly due to an increased training dataset (Figure 2). In 2020, GI Genius became available in many countries (Australia, Israel, and the UAE). In 2021, it was introduced to the United States market with an FDA de novo clearance [13], followed shortly thereafter by Health Canada approval. Lately, the number of countries in which it is available has increased further to include Singapore, India, Saudi Arabia, and Turkey.

In 2021, in addition to the already existing CADe module, a CADx module was added to GI Genius version 3 (v3), which received CE clearance under the European Medical Device Directive (MDD, 93/42/EEC) as a class IIa medical device. GI Genius CADx was utilized in a series of studies that assessed the performances of its novel feature for polyp characterization [18,19,20].

### 2.2. Video Flow for Real-Time Augmentation

The human visual system can process 10 to 12 images per second and perceive them individually, while higher rates are perceived as motion [32]. The sample rate of an endoscopy video is usually set four to six times above this threshold, at between 50 and 60 images per second. Therefore, the input for GI Genius can be imagined as an infinite sequence of individual images that are separated by a fixed interval. For the sample rates between 50 and 60 Hz, each image persists on a display for a time varying between 20 and 16.7 ms.

The task for GI Genius is to avoid introducing any delay to the flow of image frames that could be perceived as disturbing by the physician or could create a safety issue for the patient. For this reason, when the video flow enters GI Genus, it is immediately split into two different streams by a dedicated video card.

An unaltered version of the input image is transmitted directly to the output with an unperceivable delay of 1.52 μs, i.e., thousands of times less than the time needed by the brain to perceive an image. Then, the AI algorithm receives the input image through a second path, and, after appropriate computation, presents an overlay to the output if a polyp is detected within the image. The time needed by GI Genius v3 to perform this computation is about 10 ms.

Since the physician uses the endoscopic video feedback to drive activities in the bowel, this configuration ensures that the original image flow conveying the medical information to the endoscopist that is observing the display is presented without delay or alteration, thus ensuring patient safety in all conditions and operations. At the same time, GI Genius can still overlay its annotations on the image frame immediately following the one used for the AI computation, providing additional real-time information to the physician to be integrated seamlessly into the medical workflow.

### 2.3. Different Neural Networks for Different Needs

The CRC screening and surveillance colonoscopy clinical workflow during a typical diagnostic procedure can be envisioned as a temporal succession of different steps (Figure 1). AI can aid the physician during these steps with varying algorithm architectures.

During the colon navigation, the CADe algorithm will highlight each region that resembles a polyp in the displayed image. The category of the AI neural networks that are aimed at finding objects within images or videos is named object detectors. Object detection is a very active area for research on computer vision. The GI Genius CADe module was inspired by the new generation of “few-shots” neural architectures [33] that are optimized for the specific challenges of polyp detection.

After detecting a polyp, the endoscopist inspects the mucosa to describe the lesion and choose an appropriate clinical course of action. In cases where the endoscopist consistently frames a polyp, the GI Genius CADx is triggered automatically, and the histology prediction is added to the green box. The GI Genius CADx module is an image classifier [11], and the associated online aggregation algorithm is optimized to output the temporal average of the CADx prediction.

The CADe and CADx GI Genius modules have been trained using supervised learning [9]. In supervised learning, a computer algorithm is trained using input data that has been labeled for a specific output. The model is then trained repeatedly until it can identify the underlying patterns and relationships between the input and output labels. This enables it to provide precise labeling outcomes when it is given new data that it has not seen before. Therefore, describing the data that are used for training the Ais, and the methods that are used for training and testing these same Ais, is perhaps even more important than understanding the underlying algorithm architecture [34,35,36,37,38].

## 3. CADe for Polyp Detection

The GI Genius CADe module receives an input sequence of the image frames from a live endoscopy procedure. The AI module that is responsible for this object detection belongs to the class of “few-shots” architectures [33]. The object detector continuously checks whether an instance of an object that is compatible with the appearance of a colon polyp is present in several frames, within a defined temporal window. Thousands of video-recorded colonoscopy procedures must be annotated (ground truth) to train (and test) such an object detector. In this process, every single image frame of the entire dataset is checked by specialists to identify the regions (if any) where a polyp is visible. Whenever a polyp is present in an image frame, a bounding box is drawn by the specialist around the polyp to allow the algorithm to autonomously “learn” the features that define the appearance of these colon polyps. To ensure the algorithm’s generalizability and minimize the AI bias, it is essential to use a large (and diverse) dataset for this phase to represent most of the potential situations that the device will encounter when used in the real world.

### 3.1. Dataset for AI Training and Testing

The GI Genius CADe v1 module has been trained, validated, and tested using data that were collected in the randomized controlled trial [39], “The Safety and Efficacy of Methylene Blue MMX^®^ Modified Release Tablets Administered to Subjects Undergoing Screening or Surveillance Colonoscopy” (ClinicalTrials.gov registry No. NCT01694966, hereafter referred to as the MB-MMX study). During the MB-MMX study, high-definition, complete-procedure (from the start of the insertion to the end of the withdrawal) colonoscopy videos were recorded with a lossless video compression. The MB-MMX study population included CRC screening and surveillance patients that were undergoing colonoscopy. The MB-MMX study had three arms: two arms in which the patients were administered with different doses of methylene blue MMX tablets, which stain the colon with methylene blue, and one arm in which the patients received a standard white-light colonoscopy (placebo).

The details of the patient demographics and dataset subdivision are summarized in Figure 2 and Table 1.

The standalone GI Genius CADe performances were assessed by using bench testing that was executed on an independent holdout set, comprising 150 full-length white-light colonoscopies. A total of 338 polyps from 105 cases were excised and subsequently sent to histology. The dataset contained 45 cases where no lesions were identified. The total number of the frames that were used for training and testing the device was over 13 million.

### 3.2. Methods for Assessing CADe Accuracy

#### 3.2.1. Sensitivity per Frame/per Lesion

A CADe device tries to detect polyps in every frame of the video flow. Consequently, the same polyp may be detected in a frame and no longer detected in a subsequent frame. In a perfect CADe, there would be detection in every single frame where a polyp is visible, so that the sensitivity per frame would be 100%.

However, suppose that the main aim of the CADe is to reduce the number of potentially missed lesions. In this case, it could be argued that it has fulfilled its purpose once the CADe has flagged a polyp, in order to direct the endoscopist’s attention toward a region of the image, even if only a single time [5,40]. Moreover, after it has been spotted, the images of a polyp from different detections, which are obtained when the endoscopist is trying to position the scope better to perform a resection, can be considered less critical. In other words, CADe detection is primarily relevant when it identifies a polyp that has just entered the field of view, before the endoscopist can detect it. In this tiny amount of time, before the lesion exits the field of view, the device must frame the potential lesion to alert the endoscopist and minimize the chances of it being missed. Therefore, a study was conducted using the holdout test set with the GI Genius CADe v1 to establish when the lesion is in this critical time frame [14]. In this study, a panel of five expert endoscopists reviewed all the video clips that contained the holdout test set polyps and recorded the moment of their first polyp detection. The average detection of the endoscopists was then used to determine when the lesion was first perceivable.

In the scientific literature, the sensitivity per lesion is usually considered to be the proportion of polyps that have been detected in at least a single frame [40,41]. Using this definition, the sensitivity of the GI Genius CADe was 99.7% and 100% for v1 and v2, respectively. However, to better capture the capabilities of the CADe to reduce the missed lesions, it might be better to consider a more conservative definition, such as the proportion of polyps for which the CADe can detect, at or before the average endoscopist’s detection time.

Using the data that were collected in [14], the sensitivity per lesion that was measured for GI Genius was 82.0% [77.4–86.0] for CADe v1 and 86.5% [82.9–90.2] for CADe v2. In other words, the GI Genius CAD v2 could anticipate the average endoscopist’s detection 86.5% of the time. These results agree with the findings that have been recently reported by a top detector on a similar challenge [22].

#### 3.2.2. False-Positive Rates

Every time a CADe lays a box over a region of an image that has not been deemed to be a polyp by an endoscopist performing a procedure can be considered to be a false-positive (FP) detection [40]. The proportion of the frames with an FP over the total number of frames in a colonoscopy was measured for the GI Genius CADe v1 to be less than 1% [14]. Although this is a meager FP rate, given the difficulty of the detection task, a clinician would be more interested in understanding if these FPs are disturbing the procedure. A recent study attempted to address this question [23], classifying the FPs of the GI Genius CADe v1 according to the subjective perceived clinical relevance and frequency in a real-life scenario. The authors found that the endoscopists discarded almost all the FPs (over 95%), without additional withdrawal time. There were 1.6 relevant false-positive results per colonoscopy; however, this translated to an extra withdrawal time of less than 10 s, accounting for roughly 1% of the total withdrawal time.

Although this subjective identification of “clinical relevance” is interesting, it is challenging to move it to a more objective definition, since there is no consensus for what an FP in a CADe-enhanced colonoscopy is [40]. Recent clinical studies on CADe have used a variety of FP definitions, ranging from time thresholds of >1 or 2 s for an incorrect alert box, to vague descriptions such as a non-polyp area that is “continuously traced by the system,” while some studies have not specified the definition of an FP at all [42].

A less subjective definition would consider the time persistence of the alert boxes based on the different threshold definitions for the FP alarms. Table 2 shows how the detection persistence over time (the duration of the detection for the same target) correlates with the lesion-level sensitivity and the number of FP targets. In addition, the statistical testing considered the repeated marking of overlays on the same target (polyps and FPs) to be a single statistical event, instead of assuming every marking in every frame to be a single statistical event.

As seen in Table 2, CADe v2 significantly improved the number of FPs that lasted less than 500 ms, thus limiting the “distraction” effect that the rapidly “appearing and disappearing” FPs may have on an endoscopist. Although the number of FPs lasting more than 500 ms increased, in terms of the number of FPs per patient, this equates to no more than one additional FP per patient.

The performances of the different CADe modules, with regard to FPs, can be more easily and objectively compared when using this approach. It could be acceptable to only consider the clusters that are longer than a given time threshold as the FPs, as suggested by Holzwanger and co-authors [42]. In this case, the performances of the GI Genius CADe are in agreement with the subjective results of Hassan et al. [23], corresponding to 1 to 2 FPs per colonoscopy, both easily identified by the endoscopist as such, without any effect on the withdrawal time.

**Figure 3:**
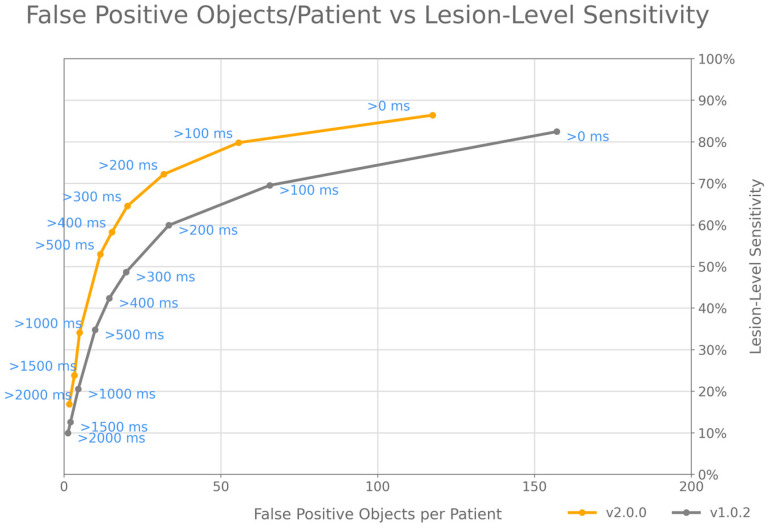
fROC analysis has to be extended to describe lesion-level sensitivity and cluster-based FP object definitions, to compare the performances of different CADe modules for polyp detection. The fROC curves displayed in the figure show the performances of GI Genius CADe v1 and v2 on the holdout test set used in Hassan et al. [14].

Considering all of the above, we propose a combination of a cluster-based FP definition and per-lesion sensitivity (Figure 3) as the best method to assess the standalone performances of the CADe algorithms for real-time polyp detection, similar to the “detection latency” method that was initially proposed by Tajbakhsh et al. [43].

## 4. CADx for Polyp Characterization

The ability to differentiate between adenomatous and non-adenomatous polyps in real time, through visual means (optical characterization) during a colonoscopy, is significant in clinical settings. It allows for the selection of the appropriate treatment protocols, prevents improper endoscopic resections, enhances cost effectiveness, and reduces the need for polypectomies [44,45]. In addition, AI has an incredible potential to support physicians in performing optical characterizations, thereby helping to standardize a medical task for which different studies have reported significant intra- and inter-rater variability [38,39].

The earlier AI algorithms for the use of CADx in colonoscopy had certain drawbacks that made it difficult to incorporate them smoothly into clinical workflows. For instance, they could only classify still images rather than videos [46], or required additional technologies, such as virtual chromoendoscopy or endocytoscopy, to function [47]. The GI Genius CADx module has been designed to work on unaltered conventional white-light video streams and to engage and disengage automatically, in order to overcome such limitations.

When performing optical characterization during a colonoscopy, the endoscopist evaluates several consecutive frames before making a decision. The GI Genius CADx seeks to emulate this decision making process by generating a temporally weighted decision for each detected polyp on a frame-by-frame basis, once it has accumulated sufficient confidence in its prediction. The subsequent paragraphs delve into the specifics of the GI Genius CADx algorithm structure and discuss the training and studies that have been conducted to evaluate its performance.

### 4.1. CADx Architecture

The GI Genius CADx module is composed of two online algorithms that work on the output of two convolutional neural network models. The first network is called the polyp characterization network, which has two main purposes: to classify each polyp as either “adenoma” or “non-adenoma” in a single video frame, and to provide an image descriptor for each detected polyp in the current frame, which is to be used for polyp tracking. The second network is the polyp imaging quality network, which provides an imaging quality score for each polyp in the current video frame. This network is essential, because low-quality images can introduce noise into the CADx’s spatial–temporal reasoning.

The first online algorithm is responsible for polyp tracking across multiple frames. In contrast, the second is an online temporal aggregation algorithm, which aggregates the frame-by-frame classification and imaging quality information for each tracked polyp and provides a live decision based on a moving temporal window. The CADx is designed to engage automatically when a polyp is consistently framed, allowing it to track and characterize different polyps simultaneously within an image frame.

The online temporal aggregation algorithm displays the live characterization decisions for each visible polyp within each frame. It can produce four different types of predictions: “adenoma”, “non-adenoma”, “no-prediction”, or “analyzing.” When the minimum number of frames is reached, a choice is displayed based on a simple majority between the “adenoma”, “non-adenoma”, and “no-prediction” classes over the number of frames for the same polyp in a fixed time interval. Since this is a moving time window, the prediction of the CADx is continuously updated based on the information of the most recent frames. The “analyzing” message is displayed when more frames are needed to decide. The CADx also automatically disengages when the standard navigation is resumed.

### 4.2. Details of AI Training and Polyp Classification

As for the GI Genius CADe v1 and v2, the polyp characterization network and the polyp imaging quality network were trained using data that were extracted from the MB-MMX study. The histopathological evaluation was based on the revised Vienna classification of gastrointestinal epithelial neoplasia [48]. The polyps that corresponded to the Vienna categories 1 (negative for neoplasia) or 2 (indefinite for neoplasia) were considered to be “non-adenoma.” On the other hand, the polyps corresponding to the Vienna categories 3 (low-grade mucosal neoplasia), 4 (high-grade mucosal neoplasia), or 5 (submucosal invasion of neoplasia) were considered to be “adenoma.” As a result of this classification, the GI Genius CADx considers all adenomatous lesions to be “adenoma”, while it considers both hyperplastic polyps and sessile serrated polyps to be “non-adenoma” [49].

**Figure 4:**
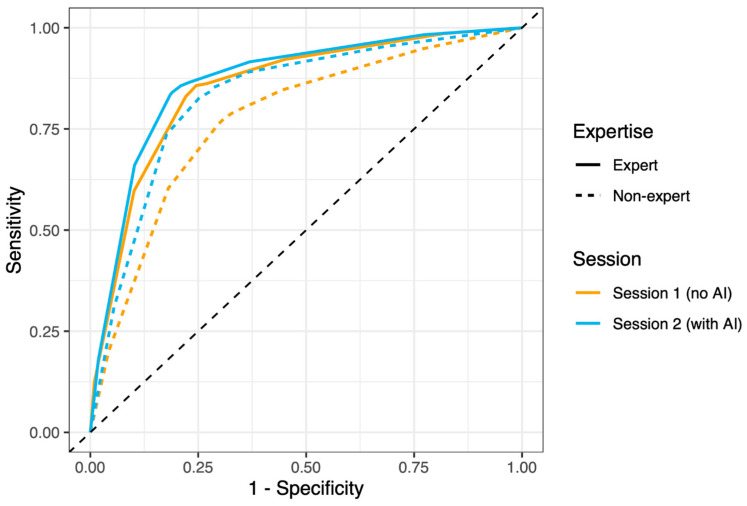
ROC curves for optical characterization performed by experts and non-experts, with and without the help of GI Genius CADx. Solid lines represent experts, dotted lines represent non-experts, orange color represent the session without AI, and the blue color represents the session with AI. The figure is from the supplementary materials of Reverberi et al. [20].

To prevent any potential mismatches in the histology results, we decided to exclude all the instances where multiple polyps appeared simultaneously or in close proximity from the dataset. This decision led to a reduced subset of the MB-MMX study (as shown in Figure 4) that could be used for the development of the CADx. The resulting video dataset was then divided into training, validation, and test subsets. The frames containing polyps were manually labeled by trained personnel, and the patients, polyps, and images were distributed as follows: 345/957/63,445 (training), 44/133/8645 (validation), and 165/405/26,412 (test).

### 4.3. CADx Performance Testing

The GI Genius CADx v3’s clinical performances were assessed in a prospective study [18] that demonstrated the device’s effectiveness in meeting the guidelines [50] for adopting a leave in situ strategy for diminutive rectosigmoid polyps. However, the optical characterization that was measured during the real-world usage of the CADx cannot be considered to be the result of the device alone, but rather the interpretation of the CADx output by the endoscopist performing the procedure.

For this reason, a different and parallel study was conducted to benchmark the “standalone” performances of the device against the accuracies of an international pool of endoscopists, which was subdivided into experts and non-experts [19]. The study showed that the overall accuracy of the device was 84.8% [81.3–87.8], with a sensitivity of 80.7% [74.2–85.7] and a specificity of 87.3% [83.0–90.6]. This accuracy was comparable to that which was measured for the experts, 82.2% [80.1–84.2], and superior to that which was measured for the non-experts 74.9% [72.4–77.3].

A third experiment was conducted to study the effects of the CADx output on an endoscopists’ optical characterization [20]. The endoscopists were asked to characterize a set of video-recorded polyps without an AI overlay in this experiment. Then, after a washout period, they were asked to review the same video clips again, this time with the CADx overlay. The results showed that the non-experts with the AI performed at the level of experts without the AI (Figure 4). In the session without the AI, the AUC values were 0.853 and 0.775 for the experts and non-experts, respectively. In the second session (with AI), the AUC values were 0.869 and 0.831 for the experts and non-experts, respectively.

## 5. Discussion

### 5.1. Study Design and Algorithm Transparency

GI Genius is the first intelligent device of its kind to become a widespread medical product that is available to endoscopists worldwide. Therefore, the scientific community could assess its performances independently in multiple clinical studies with different protocols [29]. Among the various results, it is worth mentioning here that the CADe feature of GI Genius was able to improve the ADR [15,17], reduce the miss rates [16], anticipate the experts in lesion detection [14,22], and boost the detection of heterogenous colon lesions [51].

The heterogeneity of the clinical validation studies that are focused on GI Genius is exceptionally relevant to addressing the complexity of evaluating the effectiveness of AI systems within real-world medical practice [9]. There is growing concern that the enthusiasm for testing novel AI algorithms in preliminary studies might overcome the indispensable need for a well-designed and statistically thorough clinical validation [34,52,53]. Indeed, the effectiveness of the GI Genius CADe in improving the ADR has been questioned [24,25,26]. However, these results have sparked an interesting scientific debate on the appropriate study design for validating the effectiveness of AI in endoscopy procedures [40,53].

In this context, we believe that it is essential to transparently report on the architecture of AI algorithms, as well as on the data that are used for training and the method that is used for testing, in order to address the concerns over the black-box nature of AI and its potential biases [34,35,36]. If vendors and the scientific community fail to communicate clearly regarding these issues, it could lead to a loss of faith and mistrust in the ability of AI to have an impact on medicine.

### 5.2. Why Is CADe Effective in Colonoscopy?

Regarding CADe in colonoscopy, it is interesting to clarify the mechanisms that make it so effective. The straightforward explanation that it helps endoscopists to find “subtle” and “hard to find” lesions is not entirely convincing. Studies where the ADR was improved [15,17] showed similar distributions of polyp morphology between the two arms of the study. If CADe were particularly effective for a specific morphology, it would significantly boost the detection of this morphology with the AI arm. Indeed, the number of diminutive polyps increased considerably with the AI arm. However, this is explained by the natural higher proportion of smaller lesions compared to larger lesions. Therefore, it appears that CADe can boost this detection regardless of shape and size. This is unsurprising when considering the brain mechanisms that are responsible for visual perception [5]. Because of the above, preclinical studies on novel algorithms should report the indicators that measure the ability of the CADe to “anticipate” the endoscopist’s detection, rather than their basic accuracy at a per-frame level [40]. Similarly, preclinical validation studies should always be conducted on endoscopy videos rather than static images, providing details on the ability of the algorithm to work in real time during an endoscopy [40,41].

To summarize, CADe has the potential to enhance polyp detection by transforming the serial search strategy that is used in conventional colonoscopy into a parallel search. This could lead to a faster and more efficient search, allowing endoscopists to devote more time and attention to improving navigation and mucosal exposure. However, it is crucial to note that the CADe is not meant to replace the human element, but rather to work in conjunction with endoscopists. The proper exposure and insufflation of the mucosa remain essential for successful detection. As with any new technology, it is essential for endoscopists to accept and integrate CADe into their workflow, in order to maximize its benefits.

### 5.3. CADx Interactive Output

The GI Genius CADx v3 prediction is based on the characterization network analyses within a fixed number of frames. However, as new images are fed into the algorithm, the earlier images are replaced like a conveyor belt, so the analyzed frames are constantly changing [19]. Thus, the GI Genius CADx can change its output interactively, based on the endoscopist moving the probe around the lesion, highlighting potential new information. The CADx overlay results from a moving average that is based on how the polyp is framed and how the endoscopist interprets the time-varying response of GI Genius. If there is not a majority of predictions for adenoma or non-adenoma, a no-prediction output will be overlaid near the polyp. This should not be interpreted as a limitation of the CADx; it is instead a design choice, which allows the endoscopist to transparently perceive the “confidence” of the AI. In fact, when the CADx associates a given polyp with a consistent label, the endoscopist will perceive the AI to be “confident” in its output. In contrast, when a “no-prediction” overlay is shown for a lesion, the endoscopist will correctly interpret the AI to be less confident. According to the findings of Reverberi et al. [20], the CADx’s ability to influence the endoscopists’ decisions was crucial, as they tended to rely more on the AI opinion when they lacked confidence and when the perceived AI confidence was high. Conversely, when the endoscopists were confident and the perceived AI confidence was low, they were more likely to stick with their diagnoses. This practice of belief revision followed Bayesian principles and contributed to the improved accuracy of the human–AI team as a whole.

The GI Genius CADx v3 has a few limitations. First, the number of polyp frames that were averaged to output a prediction was chosen so that the algorithm could consider a sufficient number of frames, in order to produce an accurate prediction while simultaneously causing only a short, affordable delay from when the polyp was first detected. However, this delay also depends on how the polyp is framed, because a quality filter will not consider images with blurred or unclear features. Therefore, for a portion of polyps, the time that is needed for the algorithm to output a decision might be perceived to be too long by the endoscopist. In general, a steady visualization of the lesion will considerably shorten the time needed for a CADx prediction. Second, the first version of the GI Genius CADx included both hyperplastic polyps and sessile serrated polyps in the non-adenoma class, according to the revised Vienna classification [48]. Although the tiny proportion of sessile serrated lesions between the diminutive (5 mm or less) rectosigmoid polyps does not affect the measured CADx performances [18,54], it is warranted for a future version of the CADx to include sessile serrated lesions as a separate output class.

## 6. Conclusions

GI Genius was the first intelligent platform to enter the endoscopy market, allowing the AI revolution to effectively reach clinical practice. Its hardware solution integrates with all endoscope brands, permitting a real-time AI with zero time delay and a seamless integration into the clinical workflow. Top-quality scientific evidence has shown that the CADe could reduce the polyp miss rates in colonoscopy, with a high sensitivity and meager FP rates. The GI Genius CADx allowed non-expert endoscopists to perform at the same level as expert endoscopists, with very high accuracies, even in conventional white light. The details of the algorithm architecture and the data that were used to train the AI device have been disclosed to the scientific community in the pursuit of a transparent AI. In the future, the novel features of GI Genius will further expand the number of applications, pathologies, and patients that benefit from AI in endoscopy, which is the first step toward an unprecedented revolution within endoscopy and CRC prevention.

## Figures and Tables

**Table 1:** Demographics and reasons for colonoscopy of the patients used for training, validation, and testing during the development of GI Genius CADe v1. The patients were derived from the international multicenter MB-MMX study (ClinicalTrials.gov NCT01694966 [39]).

	Training/Validation (568 Subjects)		Holdout Test Set (150 Subjects)		Overall (718 Subjects)	
Mean Age, years (SD)	61.6	(6.58)	61.5	(6.32)	61.6	(6.59)
Sex, N (%)						
Male	370	(65.1%)	93	(62.0%)	463	(64.5%)
Female	198	(34.9%)	57	(38.0%)	255	(35.5%)
Indication for Colonoscopy, N (%)						
Screening	270	(47.5%)	73	(46.7%)	343	(47.8%)
Surveillance ≤ 2 years	43	(7.6%)	7	(4.7%)	50	(7.0%)
Surveillance > 2 years	255	(44.9%)	70	(48.7%)	325	(45.3%)
Race/Ethnicity						
White or Caucasian	522	(91.9%)	141	(94.0%)	663	(92.3%)
Black or African American	34	(6.0%)	5	(3.3%)	39	(5.4%)
Hispanic or Latino	7	(1.2%)	0	(0%)	7	(1.0%)
Asian	3	(0.5%)	3	(2.0%)	6	(0.8%)
Native Hawaiian or other Pacific Islander	1	(0.2%)	1	(0.7%)	2	(0.3%)

**Table 2:** False-positive clusters for GI Genius CADe. The performances of different CADe modules regarding FPs can be more easily and objectively compared when using this approach. It could be acceptable to only consider the clusters that are longer than a given time threshold as FPs, as suggested by Holzwanger and co-authors [42]. In this case, the performances of GI Genius CADe are in agreement with the subjective results of Hassan et al. [23], corresponding to 1 to 2 FPs per colonoscopy, both easily identified by the endoscopist as such, without any effect on the withdrawal time.

	GI Genius CADe v1		GI Genius CADe v2	
Label	Overall FP	FP per Patient	Overall FP	FP per Patient
Bin 1: <500 ms	21,962	146.41	15,903	106.02
Bin 2: ≥500 ms <1000 ms	896	5.97	907	6.05
Bin 3: ≥1000 ms <1500 ms	283	1.89	303	2.02
Bin 4: ≥1500 ms <2000 ms	118	0.79	154	1.03
Bin 5: ≥2000 ms	187	1.25	269	1.79

## Data Availability

Not applicable.
